# League of Mercy

**Published:** 1907-12-28

**Authors:** 


### HOSPITAL ADMINISTRATION.

### CONSTRUCTION AND ECONOMICS.

### LEAGUE OF MERCY.

IMPROVED RESULTS OBTAINED, DESPITE FINANCIAL DEPRESSION.

The eighth annual meeting of Presidents of the League of Mercy was held in the Governors' Hall of St. Thomas's Hospital on Friday, December 20, Lord Farquhar presiding.

Colonel Hamilton (Hon. Secretary to the Presidents) read the minutes of the last meeting. Mr. E. W. Wallington (Hon. Registrar of the Order) read the following message from the Grand President, the Prince of Wales:—

"I am directed by the Prince of Wales to say that the Princess and he have learnt with great satisfaction that the work of the League of Mercy continues to extend, and that, while this year larger distributions are being made to the hospitals in the Home Counties, the League's contribution to the King's Fund will be the same as last year—£18,000.

"It was with sincere regret that their Royal Highnesses received the news of the untimely death of Mr. Reginald Lund, whose good work and special aptitude for the position of Organising Secretary were so well known to all those with whom he was brought in contact.

"Their Royal Highnesses are pleased to hear that arrangements have been made to invite a large number of the active members of the League to a concert in the Albert Hall next May, which, it is hoped, the King and Queen will attend, and at which several of the best artistes have most kindly volunteered their assistance.

"On behalf of the Lady Grand President and himself as Grand President, the Prince of Wales wishes again to thank the Presidents and Lady Presidents who have so successfully toiled to bring the League of Mercy to its present well-established position."

Lord Farquhar said it was his first duty to express the hearty thanks of the meeting for the kind message they had just had read. In that their Grand President had expressed the regret they all must feel at the death of Mr. Lund, who had worked as Organiser of the League of Mercy in a very masterly way. During the five years he held his office the income had gone up from under £9,000 to nearly £20,000. All who had to do with him felt his loss very much indeed. The arrangements for the great concert at the Albert Hall next May, at which it was sincerely hoped that all their Royal Highnesses would be present, were progressing very satisfactorily. They must again express their gratification at the continued interest taken in the League, not only by the Prince and Princess of Wales, but also by Princess Alexander of Teck and Princess Victoria of Schleswig-Holstein, who all of them worked for the cause they all had at heart with energy and undoubted success. Although that year had not been generally a successful one, the prevailing depression had not affected the League of Mercy. They had been able to hand over to the King's Hospital Fund the same amount as last year, and to increase the grants to the local hospitals in the Home Counties. Since their foundation they had been able to hand over in all £79,000 to the King's Hospital Fund, which had distributed no less than £750,000 to the hospitals of London, and this without any prejudice to the other funds. They had now got some 70,000 or 80,000 people interested in the League, and he thought all would agree that the results of their first nine years' working were very satisfactory, and hoped the next nine years would yield as good results.

The Right Hon. James Round then moved that Lord Farquhar be appointed Chairman; Mr. Frederick Green, Deputy Chairman; and Colonel H. B. Hamilton, Hon. Secretary, respectively, for the ensuing year, which was carried with acclamation.

Dr. Strong then moved that the following seven representative members of the Executive Committee be appointed: Lord Farquhar, Mr. R. C. Antrobus, Mr. Frederick Green, Mrs. R. L. Harrison, Mr. Edward Murray Ind, Mrs. Buxton Morrish, and Mr. Montagu Sharpe, and this also was agreed to.

Mrs. Rodwell (a Lady Vice-President of the Fulham District) gave a short address on the work of the League, which, she said, had now attained great importance as a well-developed scheme. Mistakes sometimes occurred and disappointments followed, but the League was there for good and all. The work was not a matter of sentiment—hardly of religion, except that work embracing and uniting all sects must be religious; they asked neither the creed nor the country of those who joined them, but they asked them all to follow the example of their Grand President and Lady Grand President in sharing the privilege of supporting those noble institutions which worked for the relief of the sufferings of their poorer brethren. She recommended the Presidents to learn the work that had to be done, and then, after a preliminary conference among a number of probable workers, to call a larger meeting, which would then have a greater chance of interesting those who came, and so making towards success. She thought meetings of members should be held at least twice a year and of Vice-Presidents, if possible, more frequently. She thought the Mayors of the various boroughs should be invited to take office in the League, and recommended that where Lady Presidents were away from home for any length of time that they should appoint able Hon. Secretaries to carry on the detail work. She found that Presidents often lost workers by lack of recognition or courtesy, sometimes they were not even acquainted with them; and if that extended from the Vice-Presidents to the members, how could they expect success? If the Presidents, Vice-Presidents, and Hon. Secretaries would meet fairly often to discuss the work and the difficulties to be overcome, many disappointments would be avoided. Classes and methods differed in different localities, and special consideration for each was required.

Miss Bucknill (Hon. Secretary of the Epsom-Esher with Godstone-Caterham District) read a suggestion on behalf of Princess Alexander of Teck that care should be taken in the distribution of the certificates issued to members.

Dr. Hewkley suggested that a hint might be given for the checking of hospital extravagance as indicated by the difference in the cost per capitum of £50 in Scotland as compared with £100 in London; but another President considered that the action taken by the King's Hospital Fund in that direction was quite adequate.

Sir William Bull asked if anything could yet be announced with regard to the acceptance of the offer of help from the London Pageant?

Colonel Ford thought it was not wise to group too many districts, as that prevented the appointment of Presidents for towns of considerable size.

### The Treasurer's Statement.

Sir Henry Burdett (Hon. Treasurer) then made his annual statement of the financial results of the year's work. He could not commence, he said, without expressing his sense of the loss they had sustained by the death of their most invaluable Secretary, Mr. Reginald Lund. He was one of the men who came to Sir Henry on leaving the Army and wanted congenial work. He recommended him first to Charing Cross Hospital, and after some experience there he became their Organising Secretary, and from the moment he joined them he (Sir Henry) felt he had seldom been associated with a more whole-hearted or more successful worker. He hoped everyone who could would take an interest in the Lund Memorial Fund for the sake of the two children he had left behind. As to the finances, his task that year was a pleasant one. The year that was going out had been a bad one financially for everybody. If a man began the year with a little money and moved it at all he was bound to finish worse off, and this, too, at the end of nine bad years, each worse than the last. In fact the past year had been the worst he ever remembered in forty years' experience of business. That they had done so well at such a time was proof that the principles of the League of Mercy had sunk into the minds and lives of those who were engaged in the work, and that they intended to go on with it as long as they had life and health. (Applause.)

Of the 106 districts which ought to be at work they had ninety-three working in 1906, and these had increased to ninety-nine in 1907. In previous years he had asked the Presidents to try and hurry up their returns of contributions so that his statement might be approximately a final one, and he now congratulated them on the fact that whereas last year at that time he had but sixty-eight returns, this year he had ninety-one. The contributions actually received to date amounted to £18,785, an average of £207 per district. In thirty-nine dstricts there had been an increase, making collectively £2,671, and in fifty-two districts where there had been a decrease the total falling-off amounted to £2,983, while in four there had been no change. The decrease that day was £312, but against that there were definite promises of money yet to be paid in amounting to £492, so that they might consider it a net gain of £180. He hoped, however, to receive a further £537 from the districts that had not yet sent in, in which case the gain for 1907 would amount to between £800 and £900, the estimated total being £20,126. He thought the most interesting feature of the returns was the increase in revenue from the Counties. The following counties showed an increase: Berks, Bucks, Hants, and Oxon collected £2,108 this year, as compared with £1,719 in 1906; Epsom-Esher £622, against £453 in 1906; Eye £212 (first year's work); Brighton (Hove) £200, as against £12 in 1906; Luton £177, as against £98 in 1906; North-East Sussex £150; Stowmarket £122; Biggleswade £119, as against £22 in 1906; Gravesend £99 (half a year's work); Ashford £92, as against £22 in 1906; Lowestoft £61; Hitchin £48 (first year's work); East Sussex £40 (first year's work); Saffron Walden £39, as against £18 in 1906; Sudbury £36, as against £14. Increases were shown also in the following Metropolitan divisions: South Paddington £842, against £733 in 1906; Strand £356, against £224 in 1906; Hornsey and Finchley £274, as against £22 in 1906. The following were the approximate returns from the different districts: Fulham £1,294; South Kensington £1,280; St. George's, Hanover Square, £941 (increase over 1906); East Marylebone £543 (increase over 1906); Kingston-Surbiton £532 (increase over 1906); Sevenoaks £487; Chelsea £466; North St. Pancras £460; West Marylebone £366 (increase over 1906); Hampstead £349; Dulwich (Norwood) £305 (increase over 1906); Westminster £299; Deptford (Brockley) 295; North Kensington £282; Chertsey £274; Guildford £265; Hertford £249; Chelmsford £237; Lewisham £214; Ealing £212 (increase over 1906); Uxbridge £201; Richmond £183; Romford £175 (increase over 1906); Woolwich £175; Eastbourne £168; Maldon £158; City of London £142 (increase over 1906); Horsham £139; Lambeth (Brixton) £135; Greenwich £133 (increase over 1906); Mile End £121; North Hackney £115; North Paddington £112; Croydon £107; Bermondsey £105 (increase over 1906); Reigate £101 (increase over 1906).

He would point out that the grants to the Counties had been very largely increased—by 100 per cent. The Presidents had been asked to send in the names of institutions they considered worthy of support. These were all carefully considered and visited—they had several appointed visitors—with the result that £1,006 had been awarded as representing the sum required to meet the claims of these particular institutions. Differing from Mrs. Rodwell somewhat, who thought the men ought not to be troubled about what she considered was mainly woman's work, he (Sir Henry) thought that men were just as anxious to join, and were just as zealous and active workers as the women were, as experience proved. Thus the ladies of Kensington, for instance, did not consider the men had done their fair share of the work during 1907, for the men had only raised £161, while the women had contributed £1,114. That was certainly not what it should be. If the men were properly roused in South Kensington, as Colonel Owen had roused them in Fulham, they would readily raise £600 a year. The result of the work of the League for the year 1907, so far as they could be estimated, should produce £20,116, as against £19,291 in 1906. The grant to the King's Fund would be £18,000, in addition to £1,006 to the country hospitals, making the total grants £19,006 in 1907. In 1905 their contribution to the King's Fund was £15,000, and last year they had made an extra large grant of £18,000 by taking some £1,500 out of their savings. The grants during the two years would approximately show an increase of £4,000—*i.e.*, from £15,000 in 1905 to £19,000 in 1907, or an average increase of £2,000 a year. If they could show the same growth every year they would have every reason to be satisfied. He was also glad to tell them that their expenses had decreased, being 7 per cent. less than in the previous year, though they had collected £1,000 more, and had six more working districts to provide for. He was perfectly certain that where the principle of personal service had been driven home, those who had taken up the work in this spirit would never give it up so long as they had their health. It was noticeable, too, that their work had had a great effect in stimulating other charitable organisations, and he had had communications from the United States which showed that their fame had spread to America, and that the principle of personal service was likely to exercise a strong influence over the people of that country.

The Honorary Secretaries' Report.

Sir William Collins, M.D., M.P., one of the Hon. Secretaries, said he desired to associate himself with what had been said about Mr. Lund; the Hon. Secretaries were well aware of the great services of which death had deprived them. He then described the progress which had been made by the League during the year, new districts having been opened, and increased interest awakened in those where organisations already existed. The Prince and Princess of Wales had during the last twelve months been pleased to appoint the following Presidents and Lady Presidents in the districts named :—

In London Districts.—North Kensington, H.H. Princess Louise Augusta of Schleswig-Holstein; Bermondsey, Mr. Carl Stettauer; Chelsea, the Countess of Mount Edgcumbe; Greenwich, Lord Alexander George Thynne, L.C.C.; Holborn, the Rev. Edward Canney, M.A.; Limehouse, Dr. and Mrs. Godding; Streatham, the Countess of Orford; South St. Pancras, Mr. and Mrs. George Alexander; Whitechapel, Mrs. W. Ellison-Macartney.

In Country Districts.—Ashford, the Lady Hothfield; Brighton, the Earl of Chichester; Eye, the Marquis of Graham; Gravesend, Colonel Sir Fitzroy D. Maclean, Bart., K.C.B., and the Countess of Darnley; Hitchin, Lord and Lady Strathcona and Mount Royal; Lowestoft, the Earl and Countess of Stradbroke; Rye, the Hon. T. A. and Lady Idina Brassey; Stowmarket, the Right Hon. Viscount Iveagh, K.P., and the Hon. Mrs. John Wood, of Hengrave.

The Prince and Princess of Wales entertained between 800 and 900 workers of the League at a garden-party at Marlborough House on July 8, when His Royal Highness awarded the Order of Mercy to 48 ladies and gentlemen connected with the League, bringing the total awards up to 237. Princess Alexander of Teck held two meetings in November at Claremont, in connection with the districts of Epsom-Esher with Godstone-Caterham, and Kingston-Surbiton with Wandsworth-Putney, and on December 6, at the invitation of Princess Christian and Princess Victoria of Schleswig-Holstein, a meeting was held at Schomberg House, when the Vice-Presidents and Lady Vice-Presidents of the Districts of Berks, Bucks, Hants, and Oxon (of which Her Highness is Lady President) attended. A great many drawing-room and other meetings had been held, at which addresses were given by the honorary officers and others connected with the League, among others in the following districts :—Ashford, Battersea (four meetings); Beds; Berks (two meetings); Biggleswade; Brighton-Hove (two meetings); Brixton (two meetings); Bucks (two meetings); Chelsea; Dulwich-Norwood; Ealing; East Marylebone (three meetings); East Sussex; Enfield; Eye; Epsom-Esher (with Godstone-Caterham), Greenwich; Guildford; Hants (eight meetings); Hitchin; Holborn; Hornsey and Finchley; Hythe; Limehouse; Lowestoft; Luton; Mile End; Mid-Essex (four meetings); North-East Sussex; North Hackney; Oxon (three meetings, one being at Nuneham Park, Oxford, at the invitation of Mrs. Harcourt, when Princess Victoria of Schleswig-Holstein was present); Peckham; Reigate; Saffron Walden; South Kensington; South Paddington (two meetings); Stowmarket; Watford; West Marylebone (two meetings); West St. Pancras; Westminster; Whitechapel. A fête in connection with the League, held in July at Park Place, Henley-on-Thames, by the kindness of Sir William Portal, when Princess Victoria of Schleswig-Holstein was present, resulted in a substantial sum being allocated to the Berks District, and £166 14s. 6d., each being handed over by Her Highness to the Royal Berkshire Hospital and the Radcliffe Infirmary, Oxford. An afternoon concert, promoted by the Lady Presidents, had been held at Bridgwater House, St. James's (kindly lent for the occasion by the Earl and Countess of Ellesmere), on July 2, which had resulted in a profit of £143 15s. Mrs. D'Oyly Carte had kindly given a matinée of "Iolanthe" at the Savoy Theatre which had proved a great success, and through the courtesy of Mrs. Gatty two matinées arranged by Mrs. Wordsworth and Mrs. Hentschel had been held at the Vaudeville Theatre. Arrangements had been made by ladies in several of the districts for a fancy-dress dance to be held at the Hôtel Cecil on January 13, 1908. A children's fancy-dress dance, held at the Hôtel Cecil in January, had resulted in a profit of £217 18s. 7d. being handed over to the funds of the League. A successful series of "Shakespearean Episodes" were arranged by several ladies and gentlemen in June, and held at Lowther Lodge (by kind permission of the Hon. William and Mrs. Lowther).

Awards to Hospitals, 1907.

With the sanction of the Prince of Wales (Grand President) the following grants for 1907 were made to Extra-Metropolitan Hospitals :—Berkshire : Royal Berkshire Hospital, Reading, £100; Windsor and Eton Royal Hospital and Dispensary, £25; St. Luke's Home, Woodley, Reading, £10. Buckinghamshire : Royal Bucks Hospital, Aylesbury, £50; Marlow Cottage Hospital, £5. Essex : Essex and Colchester General Hospital, £21; Romford Cottage Hospital, £10; Chelmsford and Essex Infirmary, £10; Brentwood District Cottage Hospital, £10; Brentwood Convalescent Home, £10; Clacton and District Hospital, £5. Hampshire : Royal South Hants Hospital, Southampton, £200; Royal Hants County Hospital, Winchester, £100; Fleet Cottage Hospital, £5. Kent : West Kent General Hospital, Maidstone, £25; Ashford Cottage Hospital, £15; Livingstone Cottage Hospital, Dartford, £10; Faversham Cottage Hospital, £10; Victoria Home for Invalid Children, Margate, £10; Hospital for Children with Hip Disease, Sevenoaks, £10; Beckenham Cottage Hospital, £10; Chislehurst and Cray Cottage Hospital, £10; Tunbridge Wells Eye, Ear and Throat Hospital, £5; Queen Victoria Cottage Hospital, Tonbridge, £5; Queen Victoria Cottage Hospital, Herne Bay, £5; Holmsdale Cottage Hospital, £5. Middlesex : Enfield Cottage Hospital, £15; Hounslow Hospital, £10; Hanwell Cottage Hospital, £5; Hayes Cottage Hospital, £5. Oxfordshire : Radcliffe Infirmary, Oxford, £100. Suffolk : Mildenhall Cottage Hospital, £5. Surrey : Royal Surrey County Hospital, Guildford, £50; Croydon General Hospital, £20; Reigate and Redhill Cottage Hospital, £15; Victoria Cottage Hospital, Woking, £10; Cranleigh Village Hospital, £10; Surbiton Cottage Hospital, £10; Egham Cottage Hospital, £10; Epsom and Ewell Cottage Hospital, £5; Caterham Cottage Hospital, £5; East and West Molesey Cottage Hospital, £5; Sutton Hospital, £5; Victoria Cottage Hospital, Leatherhead, £5. Sussex : Sussex General County Hospital, Brighton, £10; Princess Alice Memorial Hospital, Eastbourne, £10; Lewes Dispensary and Infirmary, £10; Petworth Cottage Hospital, £5.—Total, £1,006.

Replying to Dr. Hewkley's remarks, Sir William reminded the meeting that the economies instituted at 16 of the great hospitals at the instance of the King's Fund had resulted in savings of £20,000 annually. In answer to Sir William Bull, he said the proposal about the Pageant would come before the Executive at its next meeting. With regard to Colonel Ford's remarks about grouping, that matter was engaging attention, but so far it had not been found satisfactory when compared with the results in Colonel Ford's district, for example; in fact, the system Colonel Ford criticised had resulted in some £2,000 received this year from a group of Counties.

The suggestion made by Miss Bucknill at the instance of the Princess Alexander of Teck would receive attention.

Votes of thanks to Sir Ernest Cassel, for the loan of his private orchestra; to St. Thomas's Hospital, for the use of the hall; and to the Chairman, for presiding, brought the proceedings to a close.

